# Antioxidants in Menopausal Transition and Male Late-Onset Hypogonadism for the Prevention of Diabetes

**DOI:** 10.3390/antiox15060659

**Published:** 2026-05-24

**Authors:** Maria Karaflou, Athina Kaprara, Dimitrios G. Goulis

**Affiliations:** 1 Endocrinology, Diabetes and Metabolism Private Practice, Neo Psychiko, 15451 Athens, Greece; 2 Unit of Reproductive Endocrinology, First Department of Obstetrics and Gynecology, Medical School, Aristotle University of Thessaloniki, 54124 Thessaloniki, Greece; athinakaprara@auth.gr (A.K.); dgg@auth.gr (D.G.G.)

**Keywords:** antioxidants, menopause, late-onset hypogonadism, transition, diabetes prevention

## Abstract

In the modern day, despite advances in medicine and the prolongation of life expectancy, the age of menopause and male late-onset hypogonadism remains the same. This narrative review describes the physiology of the human reproductive system in females and males based on clinical and experimental studies. It explores the impact of gonadal aging and reproductive hormone withdrawal on the development of insulin resistance and diabetes and summarizes the use of antioxidants for the prevention of diabetes during menopausal transition and male late-onset hypogonadism. Maintaining high antioxidant capacity in these periods prevents the metabolic consequences of oxidative stress and improves health span trajectories. In clinical practice, we conclude that antioxidants should be used with caution to avoid the ‘antioxidant paradox’.

## 1. Introduction

Menopause and male late-onset hypogonadism (LOH) are well-described transitions in females and males during their lifespan. Experimental data and clinical studies highlight the cardiometabolic changes associated with gonadal aging and reproductive hormones’ withdrawal [1]. Body composition changes with increased fat mass and abdominal fat distribution are associated with insulin resistance and subsequent diabetes in older ages [1,2]. In this narrative review, we will explore the causative relation between oxidative damage and the metabolic consequences of reproductive hormones’ withdrawal. We also elaborated on the use of antioxidants as a preventative measure against these metabolic consequences. We consulted peer-reviewed publications on www.pubmed.gov on 1 March 2026 using the following keywords: antioxidants 1; menopause 2; late-onset hypogonadism 3; transition 4; and diabetes prevention. Experimental and clinical studies, as well as reviews published between 2010 and 2026 in the English language, were selected for citation.

## 2. Physiological Redox Signaling vs. Oxidative Damage: Balance vs. Imbalance

Reactive oxygen and nitrogen species (ROS/RNS) are continuously generated in aerobic cells as byproducts of metabolism and by dedicated enzymatic systems, such as NADPH oxidase, nitric oxide synthase, and the mitochondrial electron transport chain. Their biological effects span a spectrum, ranging from physiological redox signaling to oxidative damage, depending on concentration, localization, duration of exposure, and the capacity of antioxidant systems.

Physiological redox signaling refers to the controlled, reversible modification of biomolecules—primarily proteins—by low concentrations of ROS/RNS. Hydrogen peroxide (H_2_O_2_) is a central signaling molecule due to its relative stability and ability to diffuse short distances. It selectively oxidizes redox-sensitive cysteine residues within proteins, forming reversible modifications, such as sulfenic acids or disulfides. These changes can alter enzyme activity, protein–protein interactions, and subcellular localization, thereby regulating signaling pathways involved in proliferation, differentiation, metabolism, and stress responses.

Reactive Oxygen Species (ROS) production is often compartmentalized, and antioxidant systems (e.g., glutathione, peroxiredoxins, and catalase) rapidly buffer ROS levels to maintain signaling specificity. Importantly, these modifications are typically reversible, allowing dynamic control, analogous to phosphorylation-based signaling networks.

In contrast, oxidative damage occurs when ROS/RNS production exceeds the buffering capacity of antioxidant defense systems, resulting in oxidative stress. Under these conditions, ROS react non-specifically with cellular macromolecules, leading to largely irreversible chemical alterations (e.g., DNA damage, protein oxidation, and lipid peroxidation). At low, regulated levels, ROS function as essential signaling mediators; at high, unregulated levels, they become agents of cellular damage [3].

These changes disrupt cellular homeostasis and are implicated in aging and the pathogenesis of numerous diseases, including cancer, neurodegenerative disorders and cardiovascular conditions.

## 3. Oxidative Stress as a Causal Mechanism of Insulin Resistance and Diabetes

It is now widely accepted that excessive ROS generation not only results from metabolic dysfunction but also plays a direct causal role in impairing insulin signaling pathways and promoting metabolic disease [4].

At the cellular level, oxidative stress disrupts insulin signaling by modifying key components of the insulin receptor cascade. Under physiological conditions, insulin binding activates the insulin receptor and downstream signaling through insulin receptor substrate (IRS) proteins, phosphatidylinositol 3-kinase (PI3K), and Akt, ultimately facilitating glucose uptake via glucose transporter 4 (GLUT4) translocation. However, ROS overproduction activates stress-sensitive kinases, such as c-Jun N-terminal kinase (JNK) and p38 Mitogen-Activated Protein Kinase (MAPK), which induce serine phosphorylation of IRS proteins, thereby inhibiting normal insulin signaling and reducing glucose uptake. This molecular interference provides a direct mechanistic link between oxidative stress and insulin resistance [4].

In addition to impairing signaling pathways, oxidative stress contributes to metabolic dysfunction by damaging cellular macromolecules. DNA damage, protein oxidation, and lipid peroxidation alter cellular function, impair energy metabolism, and cause chronic inflammation. These effects are particularly evident in insulin-sensitive tissues, such as the skeletal muscle, liver, and adipose tissue, where oxidative damage disrupts glucose homeostasis and promotes insulin resistance. Furthermore, mitochondrial dysfunction—both a source and a consequence of ROS production—exacerbates this process, creating a self-perpetuating cycle of oxidative stress and metabolic impairment [5].

Oxidative stress is closely associated with conditions that predispose individuals to T2DM, including obesity. Elevated levels of glucose and free fatty acids increase mitochondrial ROS production and activate metabolic pathways (e.g., protein kinase C activation and formation of advanced glycation end-products), contributing to insulin resistance and β-cell dysfunction. Over time, these processes impair pancreatic β-cell function, reducing insulin secretion and accelerating the transition from insulin resistance to overt diabetes [5]. Oxidative stress is linked not only to type 2 diabetes but also to diabetes-related complications [6,7].

## 4. Female Reproductive System, Oxidative Stress, and the Menopausal Transition

The ovaries play a dual role in female reproduction, namely, steroidogenesis and folliculogenesis. They are under the control of gonadotropin-releasing hormone (GnRH), secreted by the hypothalamus, and luteinizing hormone (LH) and follicle-stimulating hormone (FSH), secreted by the pituitary. Sex steroids produced by the ovaries exert a negative feedback effect on the hypothalamus and pituitary, which are components of the hypothalamic-pituitary-gonadal axis [8,9].

### 4.1. Ovarian Steroidogenesis

During the reproductive years, hormone production in the ovary occurs in the maturing follicle. The follicles had the following components:

- Theca cells, producing androgens;
- Granulosa cells, producing estrogens;
- Primary oocyte containing genetic material.

In menopause, when females are depleted of mature follicles, other cell types, such as stromal cells (secondary interstitial and hilum cells), continue to produce androgens, but only modestly [10]. Ovarian androgen production presents a gradual decline through the menopause transition [10,11,12], whereas the adrenal glands become the main source of androgens [13].

Ovarian hormones are derived from cholesterol. Steroidogenic cells acquire the cholesterol substrate mainly from low-density lipoprotein (LDL). Stimulation of ovarian cells by the trophic hormones FSH and LH facilitates the uptake of cholesterol by increasing the number of LDL receptors on the cell surface. LDL particles are subsequently internalized and degraded in the lysosome. Free cholesterol is then transferred into the mitochondria by the steroidogenic acute regulatory protein (StAR), whose action is a rate-limiting step determining substrate availability for steroidogenesis.

Cholesterol is converted into pregnenolone, the precursor of all steroid hormones, in mitochondria by the P450scc enzyme, which regulates cholesterol side chain cleavage. Subsequently, pregnenolone is transported from the mitochondria to the smooth endoplasmic reticulum, where the remaining steps of steroidogenesis occur. Ovarian cells secrete different hormones due to differential enzyme activity. Theca interstitial and secondary interstitial cells lack aromatase and are, therefore, androgen-producing cells of the ovarian cortex, mainly producing androstenedione. Granulosa cells express the necessary enzymes for the aromatization (by P450 aromatase) of androstenedione to estrone and for its subsequent reduction to estradiol (by 17β-hydroxysteroid dehydrogenase 1). Each regulatory step may be susceptible to oxidative stress [14,15]. Zaidi et al. have shown that superoxide dismutase-2 deficiency-induced oxidative stress attenuates steroidogenesis in mouse ovarian granulosa cells, leading to decreased progesterone and estradiol levels. The underlying mechanism is the reduced expression of the StAR protein and other steroidogenic enzymes [15].

### 4.2. Folliculogenesis and Ovarian Aging

Human follicles begin development in the fourth gestational month, when approximately 1000 to 2000 germ cells begin to migrate to the gonadal ridge and multiply, reaching 6 to 7 million by the fifth month of gestation. Even at this point, the number of follicles begins to decline, and female neonates have approximately 1 million follicles at birth. In adult females, follicles may remain quiescent, be recruited for further development and ovulation, or be destroyed via apoptosis.

In reproductive years, menstrual cycles are preserved and they exhibit two phases:

- The follicular phase characterized by the growth of the dominant follicle and ovulation;
- The luteal phase following ovulation, which includes the formation of the corpus luteum and the secretion of progesterone.

The menopausal transition is characterized by a gradual loss of oocytes, altered responsiveness to gonadal steroid feedback, and hormonal fluctuations with a gradual decline in serum estrogen concentrations [16]. The menopausal transition begins at the same time as perimenopause but ends with the last menstrual period [16]. Chronological and ovarian aging are two concurrent processes that influence the pace and duration of perimenopause [16].

### 4.3. Hormonal Withdrawal, Oxidative Stress and Subsequent Metabolic Consequences in Menopausal Transition

Alterations in hormonal status lead to incomplete reduction of oxygen and subsequent ROS formation [17]. Increased oxidative stress has been associated with severe menopausal symptoms [18,19] and is linked to gonadal aging [15]. Sanchez-Rodriguez et al. showed that lipoperoxide levels were significantly higher in postmenopausal women than in the premenopausal group and that menopause was a risk factor for oxidative stress by using logistic regression analysis to control pro-oxidative variables [18]. The Menopause and Oxidative Stress Project showed a strong correlation between the severity of hot flashes and oxidative stress estimated by measuring plasma malondialdehyde using the TBARS assay, erythrocyte superoxide dismutase (SOD), glutathione peroxidase (GPx), uric acid, and total antioxidant status [19]. Unfer et al. evaluated the behavior of blood antioxidant enzymes SOD, catalase, and glutathione peroxidase, plasma total antioxidant capacity, and oxidative damage (lipid oxidation and protein carbonyl levels) following estrogen plus progestin treatment in postmenopausal women and found that total antioxidant capacity increased [20].

Perimenopause leads to increased adiposity and abdominal fat distribution. On average, women gain 2–3 kg during the menopause transition, with high interindividual variability [21]. Marlatt et al. showed that Caucasian perimenopausal women presented increased total and abdominal adiposity (+6% in body fat percentage, +9% in fat mass, +12% in trunk fat mass, +19% in abdominal subcutaneous fat, and +15% in visceral fat) and simultaneous increases in total cholesterol and LDL-cholesterol during the menopausal transition [22]. Visceral fat distribution is harmful, as a fatty liver induces insulin resistance and predisposes to diabetes [1,23]. Both estradiol and FSH regulate energy balance [1]. Liu et al. have shown that blocking FSH leads not only to increased bone mass, but also to a remarkable reduction in adiposity, coupled with the production thermogenic adipose tissue [24]. Estradiol also affects numerous energy homeostasis pathways, that is, the control of appetite, energy expenditure, regulation of lipid storage, and sensitivity to insulin [1,2].

**Figure 1:**
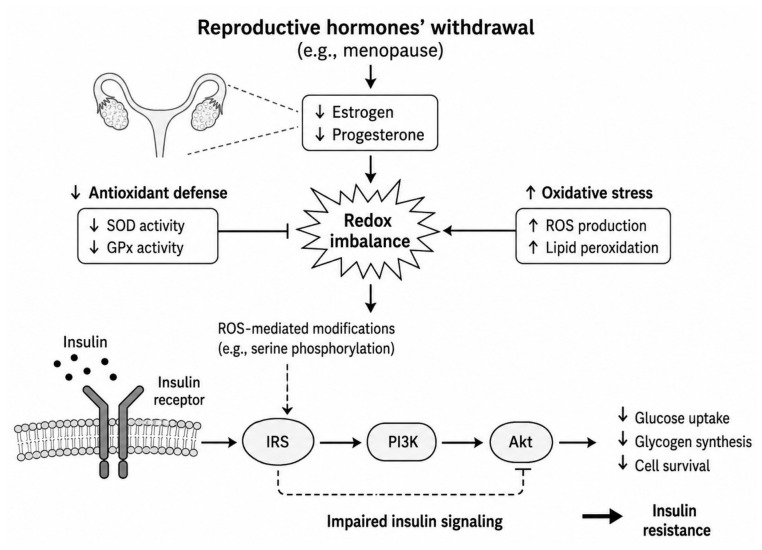
Withdrawal of reproductive hormones constitutes the basis of redox imbalance and subsequent insulin resistance. SOD: superoxide dismutase, GPx: glutathione peroxidase, ROS: reactive oxygen species, IRS: insulin receptor substrate, PI3K: phosphoinositide 3 kinase, Akt: protein kinase B.

Perimenopause has also been linked to decreased muscle mass and bone mass [2]. Steyn et al. showed that increased central fat deposition is associated with reduced plasma antioxidants, and that this reduction contributes to the risk of cardiorenal disease [6] (Figure 1).

## 5. Male Reproductive System, Oxidative Stress, and Late-Onset Hypogonadism

Male late-onset hypogonadism is not an inevitable outcome of aging but a clinical condition in older men characterized by low serum testosterone levels accompanied by relevant clinical symptoms in the absence of an organic disorder affecting the hypothalamic-pituitary-testicular (HPT) axis [25]. Its pathophysiology is closely linked to metabolic and molecular alterations that accompany aging.

### 5.1. Testicular Steroidogenesis and Spermatogenesis and Oxidative Stress

Oxidative stress disrupts male reproductive function primarily by interfering with the hypothalamic–pituitary–gonadal axis. Elevated ROS levels impair hypothalamic signaling, leading to reduced GnRH release and subsequent suppression of LH and FSH secretion, ultimately decreasing testosterone production and reducing spermatogenesis. In parallel, oxidative stress promotes the production of inflammatory cytokines (e.g., TNF-α, IL-1β, IL-6), which further inhibits hormonal regulation. Additionally, activation of the hypothalamic–pituitary–adrenal axis increases cortisol levels, which suppresses LH secretion and exacerbates testosterone deficiency [26].

At the testicular level, oxidative stress directly impacts Leydig cells responsible for testosterone synthesis. ROS-induced damage includes lipid peroxidation of cellular membranes, protein denaturation, and DNA fragmentation, all of which compromise cellular integrity and function [27]. Moreover, oxidative stress impairs essential steroidogenic enzymes involved in testosterone formation, thereby diminishing hormone production [27]. Furthermore, ROS interfere with the communication between Leydig cells and the HPT axis, hindering the coordinated regulation of testosterone production [26]. Sertoli cells, which support sperm production and regulate the microenvironment around seminiferous tubules, are highly vulnerable to oxidative damage. ROS and environmental stressors can interfere with Sertoli cell function by impairing gap junction communication, disrupting the blood–testis barrier, and modifying hormonal signaling [27,28]. These changes impair spermatogenesis and decrease the synthesis of androgen-binding protein (ABP), which is necessary to sustain elevated levels of intratesticular testosterone [26].

In addition to its direct effects on cells, oxidative stress affects intragonadal regulatory networks involving paracrine and autocrine factors. Growth hormone (GH), insulin-like growth factor-1 (IGF-1), prolactin, inhibin, and estrogen are among the hormones crucial for controlling spermatogenesis and testosterone synthesis [29,30]. Βy increasing levels of inhibin and estradiol, oxidative stress disrupts this delicate equilibrium, as estradiol directly suppresses testosterone synthesis while inhibin B acts indirectly via negative feedback on pituitary FSH secretion [31]. Moreover, ROS can increase prolactin production, which lowers GnRH release and further suppresses the HPT axis [30].

Another important factor linking oxidative stress and male late-onset hypogonadism is inflammation. Proinflammatory cytokines, such as IL-1β, IL-6, and TNF-α, are produced when ROS levels are elevated. These cytokines adversely affect the HPT axis and inhibit the production of testosterone. This persistent low-grade inflammatory state is frequently observed in metabolic diseases and aging, strengthening the link between oxidative stress and hypogonadism [27].

Several environmental and lifestyle factors significantly contribute to oxidative stress and the development of male late-onset hypogonadism. Increased ROS production and impaired reproductive function are linked to pesticide exposure, air pollution, radiation, endocrine-disrupting chemicals, smoking, alcohol use, obesity, and psychological stress [27]. For example, it has been demonstrated that pesticides, such as diazinon, cause oxidative stress, interfere with steroidogenic pathways, and lower testosterone levels by downregulating important hormone synthesis-related enzymes and disrupting Leydig cell function [32].

Furthermore, oxidative stress impacts vascular function by the interaction of ROS with nitric oxide (NO), resulting in the production of peroxynitrite and diminished vasodilatory ability. This leads to endothelial dysfunction, a fundamental mechanism underlying erectile dysfunction in men with late-onset hypogonadism [33]. The decline in testosterone levels exacerbates this process by affecting nitric oxide synthase (NOS) activity, thereby establishing a harmful cycle among oxidative stress, hypogonadism, and vascular dysfunction [27].

In summary, oxidative stress disrupts the HPT axis, impairs the function of Leydig and Sertoli cells, modifies endocrine control, and increases inflammation, all of which contribute to the pathophysiology of late-onset male hypogonadism. Its effects extend beyond hormonal imbalance and include impaired reproduction and vascular malfunction. Developing focused treatment approaches to reduce oxidative stress and enhance outcomes in men with age-related hypogonadism requires an understanding of these mechanisms.

### 5.2. Hormonal Withdrawal, Oxidative Stress and Subsequent Metabolic Syndrome in Male Late-Onset Hypogonadism

Late-onset hypogonadism is more prevalent in elderly men with comorbidities, including metabolic syndrome, obesity, and type 2 diabetes. Men experience a gradual and variable decline in testosterone, in contrast to women during menopause [34]. Hypogonadism and reduced testosterone levels are associated with increased oxidative stress through impairment of systemic antioxidant defenses. In particular, men with low testosterone exhibit decreased levels of key antioxidants such as coenzyme Q10 and reduced total antioxidant capacity, indicating a shift toward a pro-oxidant state [35]. Moreover, evidence indicates that low testosterone is not merely a consequence of metabolic disease but may also contribute directly to the development of insulin resistance, establishing a bidirectional relationship [36]. A key factor in this relationship is visceral adiposity. Several studies have suggested that central obesity, rather than testosterone levels per se, is the primary determinant of insulin sensitivity after adjusting for confounders [37].

Mechanistically, insulin resistance contributes to hypogonadism through multiple pathways. At the central level, men with insulin resistance exhibit impaired dynamic responses of the HPT axis, including reduced secretion of LH, FSH and testosterone following stimulation with GnRH [38]. At the testicular level, insulin resistance appears to impair Leydig cell function directly. Clinical studies have demonstrated reduced baseline and stimulated testosterone production in men with type 2 diabetes and increased waist circumference [39].Additionally, an inverse relationship has been observed between insulin resistance and testicular responsiveness to hormonal stimulation, such as human chorionic gonadotropin (hCG) [40]. Oxidative stress on the testis and directly on germ cells is the main cause of male infertility in patients with diabetes [41].

Conversely, testosterone plays an active role in glucose regulation. It enhances β-cell function and insulin secretion, partly via incretin pathways, such as glucagon-like peptide-1 (GLP-1). Additionally, testosterone improves insulin signaling and glucose uptake in the adipose tissue and skeletal muscle, the main sites of insulin-mediated glucose disposal. It increases the expression of key metabolic regulators, including insulin receptor substrates, AKT, AMPK, and glucose transporters, such as glucose transporter type 4 (GLUT-4), thereby promoting glucose utilization and mitochondrial function [36].

Testosterone replacement therapy (TRT) has attracted significant interest regarding its effects on insulin resistance. Clinical studies suggest that TRT can improve insulin sensitivity, as reflected by reductions in indices such as the Homeostatic Model Assessment of Insulin Resistance (HOMA-IR), as well as decreases in fasting glucose and insulin levels [42,43,44]. Additionally, TRT appears to favorably influence body composition by reducing fat mass and increasing lean muscle mass, which may contribute to metabolic improvements [45]. Mediation analyses indicate that improvements in glucose metabolism observed during TRT are largely driven by reductions in fat mass [46]. Nevertheless, evidence of acute changes in insulin sensitivity prior to alterations in body composition suggests that testosterone may exert direct metabolic effects independent of adiposity [47]. However, findings across studies are not entirely consistent, with some trials showing minimal or no effects on glycemic control [48,49]. A more nuanced view is offered by two recent randomized controlled trials. In men with impaired glucose tolerance or newly diagnosed type 2 diabetes, two-year testosterone treatment decreased the percentage of participants with type 2 diabetes beyond the effects of a lifestyle program, according to the T4DM trial [50]. Further research is needed to determine the long-term durability, safety, and cardiovascular effects of TRT. In contrast, TRT was non-inferior to placebo in terms of the incidence of major adverse cardiac events in men with hypogonadism and a high risk of cardiovascular disease in the TRAVERSE trial. However, insufficient evidence supports improvements in glycemic outcomes [51].

Taken together, TRT may improve body composition and insulin resistance in men with obesity and type 2 diabetes; inconsistent effects on HbA_1c_ do not support its use as monotherapy. It may be considered in hypogonadal men with significant insulin resistance or diabetes, alongside lifestyle modification and standard medical treatment [25].

## 6. The Role of Antioxidants in Obesity and Diabetes

A structured, non-systematic literature search was performed in PubMed for studies published within the last decade. Combinations of Medical Subject Headings (MeSH) and free-text terms were used, including “antioxidants”, “oxidative stress”, “alpha-lipoic acid”, “resveratrol”, “vitamin C”, “vitamin E”, “type 2 diabetes mellitus”, “insulin resistance”, “obesity”, and “hypogonadism”. Boolean operators (AND, OR) were applied to refine the search. Filters were used to prioritize randomized controlled trials, systematic reviews, and meta-analyses in human populations. The reference lists of relevant articles were screened to identify additional studies. Studies not published in English, animal studies, and those lacking relevant outcome data were excluded. Selected studies were included to illustrate representative findings on antioxidant interventions in metabolic disorders rather than to provide a comprehensive or systematic synthesis of the available evidence.

Concerning resveratrol and obesity, a systematic review and meta-analysis of 28 randomized controlled trials evaluated the effects of resveratrol supplementation on anthropometric parameters in adults. The pooled analysis demonstrated modest but statistically significant reductions in body weight, body mass index (BMI), and waist circumference, whereas no significant effect was observed on fat mass. Subgroup analyses indicated that lower doses (<500 mg/day), longer intervention durations (≥3 months), and studies conducted in individuals with obesity were associated with more pronounced improvements [52]. A more recent (2026) systematic review and meta-analysis of 23 randomized controlled trials (n = 1005) evaluated the effects of resveratrol on anthropometric indices and adipokines in individuals with overweight or obesity. Overall, resveratrol did not significantly improve body weight, BMI, fat mass, or circulating adiponectin and leptin levels. A modest but significant reduction in waist circumference was observed. Subgroup analyses indicated that lower doses (<1000 mg/day) and longer intervention durations (≥12 weeks) were associated with small improvements in body weight and BMI [53]. These discrepancies between the two meta-analyses may reflect differences in study design, participant characteristics, and dosing strategies; however, they highlight that antioxidant supplementation does not consistently translate into metabolic benefits in humans.

Regarding resveratrol and diabetes, a 2022 systematic review and meta-analysis evaluated the dose- and age-dependent effects of resveratrol on glycemic control in type 2 diabetes mellitus. Resveratrol supplementation demonstrates age- and dose-dependent effects on glycemic parameters. In individuals aged 45–59 years, significant reductions in glucose levels were observed across a broad dose range (<250, 250–500, and 500–1000 mg/day), with greater reductions at higher doses. In contrast, in individuals older than 60 years, glucose lowering was evident only at intermediate doses (250–500 mg/day). Similarly, improvements in HbA_1c_ were restricted to the 45–59-year age group at doses of 250–500 mg/day, with no significant effects in older individuals. Insulin levels followed a comparable pattern, showing improvements at lower and intermediate doses in younger individuals, whereas in older adults, the benefits were limited to moderate dosing. Overall, these findings highlight substantial heterogeneity and preclude the identification of a clearly effective therapeutic dose [54]. A more recent (2024) meta-analysis of randomized, double-blind, placebo-controlled trials evaluated the effects of resveratrol supplementation on inflammation and oxidative stress in patients with type 2 diabetes mellitus. Across six RCTs (n = 533), resveratrol significantly reduced markers of oxidative stress, including lipid peroxides and 8-isoprostanes, and lowered C-reactive protein levels. It also increased antioxidant enzyme activity, such as glutathione peroxidase and catalase. However, no consistent effects were observed for key inflammatory cytokines, including interleukin-6 and tumor necrosis factor-α. Importantly, the overall quality of evidence ranged from low to very low, highlighting the need for larger, well-designed trials [55]. Overall, resveratrol demonstrates modest, dose- and age-dependent effects on oxidative stress and glycemic parameters; however, these benefits are inconsistent and preclude firm clinical recommendations.

Regarding the metabolic effects of vitamins, a 2025 systematic review and meta-analysis of randomized controlled trials evaluated the effects of vitamin supplementation on adipokine levels in adults. Overall, vitamin E did not significantly affect circulating adiponectin or leptin concentrations. However, subgroup analyses indicated that longer intervention durations (>12 weeks) were associated with increased adiponectin levels, particularly in individuals with nonalcoholic fatty liver disease (NAFLD). Additionally, a significant reduction in leptin levels was observed in this subgroup. These findings suggest that the metabolic effects of vitamin E may be context-dependent, with greater benefits observed in specific patient populations. Nevertheless, heterogeneity in study design, dosage, and duration limits the generalizability of these results [56].

Regarding vitamins and diabetes, a 2021 GRADE-assessed systematic review and meta-analysis of 28 randomized controlled trials (n = 1574) evaluated the effects of vitamin C supplementation on glycemic control and cardiovascular risk factors in individuals with type 2 diabetes. Vitamin C supplementation was associated with significant reductions in HbA_1c_, systolic, and diastolic blood pressure; however, the certainty of evidence ranged from moderate to very low. Most included studies were short-term and had small sample sizes, limiting the robustness of the conclusions. Overall, while short-term benefits were observed, current evidence is insufficient to support vitamin C supplementation as a standard therapeutic strategy [57]. A 2026 systematic review and subgroup meta-analysis of 52 randomized controlled trials (n = 1425) assessed the effects of vitamin C and vitamin E supplementation on glycemic control and cardiovascular risk factors in type 2 diabetes. Overall, both vitamins, alone or in combination, showed comparable effects on glycemic indices, diastolic blood pressure, and most lipid parameters. However, reductions in systolic blood pressure were observed only with vitamin C or combined supplementation, whereas increases in HDL-cholesterol were evident exclusively with the combined regimen. These findings suggest differential, outcome-specific effects influenced by antioxidant type and intervention characteristics [58]. Collectively, evidence from randomized controlled trials indicates that vitamin C and vitamin E exert modest and outcome-specific metabolic effects; however, these benefits are inconsistent and often supported by low-certainty evidence. Substantial heterogeneity in study design, dosing, and duration limits their clinical applicability.

Regarding alpha-lipoic acid and obesity, a 2018 systematic review and meta-analysis of 12 placebo-controlled clinical trials evaluated the effects of alpha-lipoic acid supplementation on anthropometric indices in adults. The results demonstrated statistically significant reductions in body weight and body mass index, with no overall significant effect on waist circumference. Stratified analyses suggested that reductions in waist circumference were more pronounced in individuals with metabolically unhealthy conditions than in healthy populations. Despite these findings, the magnitude of the effect was relatively small, raising questions regarding the clinical relevance and cost-effectiveness of alpha-lipoic acid in obesity management [59]. A 2020 systematic review and meta-analysis of randomized placebo-controlled trials (including 18 studies for weight, 21 for BMI, and 8 for waist circumference outcomes) evaluated the effects of alpha-lipoic acid supplementation on obesity-related indices. The analysis demonstrated significant reductions in body weight and body mass index, whereas no overall effect was observed on waist circumference. The findings indicated that intervention duration was a key determinant of waist circumference reduction, with longer interventions showing greater effects. Subgroup analyses suggested a potential sex-specific response, with more pronounced reductions in waist circumference among women [60].

A 2025 systematic review and meta-analysis of 11 randomized controlled trials (n = 704) evaluated the effects of alpha-lipoic acid supplementation on intermediate metabolic markers in overweight and obese adults. Overall, no significant effects were observed on lipid parameters, including triglycerides, total cholesterol, HDL-cholesterol, and LDL-cholesterol, nor on glycemic indices such as fasting blood glucose and insulin resistance (HOMA-IR). Despite generally low risk of bias across studies, substantial heterogeneity was noted for certain outcomes. These findings suggest a limited efficacy of alpha-lipoic acid in metabolic risk markers, highlighting the need for longer-duration trials, higher doses, and better-targeted populations [61]. Moreover, a 2026 systematic review and meta-analysis of 15 studies evaluated the effects of alpha-lipoic acid supplementation in patients with diabetic polyneuropathy. The analysis showed that the majority of evaluated outcomes favored alpha-lipoic acid, with significant improvements in neuropathic symptoms, such as paresthesia, numbness, and pain scores, particularly at a dose of 600 mg/day. However, no significant effects were observed on glycemic control (HbA_1c_), nitric oxide levels, or nerve conduction parameters. These findings suggest that alpha-lipoic acid may provide symptomatic relief, although its impact on underlying metabolic and neurophysiological outcomes remains limited [62]. Collectively, evidence from randomized controlled trials indicates that alpha-lipoic acid exerts modest and context-dependent effects, with small reductions in anthropometric indices and improvements in neuropathic symptoms, but no consistent benefits on core metabolic outcomes. This discrepancy between symptomatic and metabolic effects, together with substantial heterogeneity in study design and intervention parameters, underscores its limited and condition-specific clinical utility.

## 7. The Role of Antioxidants in Females Undergoing Menopause and in Males Undergoing Late-Onset Hypogonadism

Zinc is a component of the antioxidant defense system and is important at various developmental stages. It regulates key processes, such as cell proliferation, differentiation, and survival. Severe gestational zinc deficiency causes teratogenesis in the brain and other organs, and mild zinc deficiency has deleterious consequences on the neural stem cell pool, neurogenesis, oligodendrogenesis, and astrogliogenesis in offspring. The above evidence indicates that zinc supplementation is encouraged in women undergoing fertility treatment to prevent brain developmental deficits in the offspring [63]. Deluao et al. supported the approach of combating increased oxidative damage in the pre-implantation embryo through the addition of antioxidants [64].

Of the known antioxidants, all-trans retinoids should be used with caution, as their excessive use may diminish cell activity, induce cellular senescence, and result in cleft palate in the offspring [65]. Vitamin E has well-established antioxidant properties. However, its use should be cautious, as vitamin E toxicity is associated with an increased risk of bleeding.

In the context of male reproductive health, antioxidants may protect Leydig cell integrity and testosterone synthesis by reducing oxidative stress at the testicular level. Oxidative stress-induced Leydig cell apoptosis is an important mechanism that connects metabolic diseases and hypogonadism. It has been demonstrated that antioxidant interventions, such as curcumin nanomicelles, increase total antioxidant capacity while improving testosterone levels, lowering gonadotropins (FSH and LH), and reducing oxidative stress markers, such as malondialdehyde (MDA), C-reactive protein (CRP), and tumor necrosis factor (TNF) [66]. The beneficial effects of antioxidants on oxidative stress and testosterone production are further supported by preclinical research. In animal models of hypogonadism, several substances, such as vitamins A and C, zinc, N-acetylcysteine (NAC), lycopene, resveratrol, and numerous plant-derived extracts, have shown improvements in steroidogenesis and decreases in oxidative damage [67]. By increasing testosterone levels and decreasing adiposity, both of which are strongly associated with insulin resistance, these changes may help improve glucose metabolism.

## 8. Antioxidant Paradox

The complicated role of ROS in physiological signaling may be the reason why antioxidant supplementation does not always result in the anticipated clinical advantages, a situation known as the “antioxidant paradox” [68].

The results of the SELECT trial offer a convincing example of the “antioxidantparadox”. Selenium and vitamin E supplements did not lower the incidence of prostate cancer in either the primary or extended analyses, whereas vitamin E significantly increased risk with longer follow-up. These findings refute the oversimplified notion that reducing oxidative stress is always advantageous and instead provide credence to the idea that reactive oxygen species are physiologically necessary for cellular activation. Therefore, high-dose supplementation that disrupts this redox equilibrium may have unexpected biological effects [69,70]. Similarly, the antioxidant paradox was demonstrated by the CARET study, which showed that supplementation with beta-carotene and vitamin A not only failed to lower disease risk but also significantly increased lung cancer incidence and overall mortality in high-risk individuals [71]. By showing that antioxidant supplementation does not have consistent effects across substances or disease endpoints, the ATBC study further highlights the complexity of the “antioxidant paradox.” While alpha-tocopherol showed no benefit for lung cancer but revealed possible protective effects for prostate cancer, beta-carotene supplementation was linked to an increased incidence of lung cancer and overall mortality in this large randomized study of male smokers. These disparate results demonstrate the lack of a consistent class effect and emphasize how antioxidant activity is context-dependent, depending on tissue type, exposure to oxidative stressors such as smoking, and underlying biological conditions [72]. The above findings question the simplistic view that reduction of oxidative stress is always beneficial and reinforce the concept that ROS are essential for normal cell signaling. Therefore, under certain biological circumstances, high-dose antioxidant supplementation may interfere with vital redox-sensitive pathways and cause unexpected biological effects.

A critical issue is whether menopause and male late-onset hypogonadism, as states of hormonal decline, may represent a distinct redox context relative to the antioxidant paradox. The reduction in estrogen and testosterone has been associated with increased oxidative stress, as mentioned earlier. This raises the possibility that targeted redox modulation could be beneficial under conditions of hormonal deficiency. However, this does not justify indiscriminate antioxidant supplementation. Reactive oxygen species remain essential for cellular signaling [3], and excessive suppression may induce reductive stress and disrupt redox-sensitive pathways, potentially explaining the adverse outcomes observed in large trials [69,70,71,72]. Given the lack of large-scale randomized controlled trials specifically in these populations, current evidence supports a cautious, context-dependent approach rather than assuming that antioxidant supplementation is inherently beneficial.

## 9. Discussion

Lifestyle factors significantly contribute to the management of oxidative stress and the reduction of diabetes risk in men with andropause. Diets rich in antioxidants, including vitamins C and E, selenium, flavonoids, and omega-3 fatty acids, can boost intrinsic antioxidant defenses and reduce inflammation [23]. Consistent moderate physical activity enhances insulin sensitivity, reduces oxidative stress, and aids in weight management; however, excessive exercise may elevate ROS production [73,74]. Weight reduction is crucial, as obesity is strongly associated with increased oxidative stress and impaired reproductive function [75]. Moreover, reducing exposure to environmental and lifestyle risk factors, such as smoking, consumption of alcohol, and pollutants, can significantly decrease ROS levels and improve metabolic and reproductive outcomes [76].

The early detection of metabolic dysfunction and hypogonadism using oxidative stress indicators and hormonal profiling may enable prompt therapeutic interventions [76]. Novel therapies, including regenerative strategies such as stem cell-derived factors, have shown potential in reducing oxidative damage and restoring testicular function; however, further clinical validation is required [77] (Table 1).

Conflicting clinical data exist to support the widespread use of antioxidants in humans. Therefore, antioxidants may help prevent diabetes in men experiencing andropause; however, their use must be properly regulated with the correct dosage and clinical supervision. Ultimately, a combined strategy that includes antioxidant support, lifestyle modification, and careful clinical monitoring appears to be the most effective approach for preventing diabetes in men with andropause.

Overview of selected meta-analyses highlighting heterogeneous and context-dependent effects of antioxidant supplementation on metabolic outcomes.

SR = Systematic review, MA = Meta-analysis, BW = Body Weight, BMI = Body mass index, WC = Waist circumference, FM = Fat mass, T2DM = Type 2 diabetes mellitus, NAFLD = Nonalcoholic fatty liver disease, SBP = Systolic blood pressure, TG = Triglyceride, TC = Total cholesterol, HDL-C = High-density lipoprotein cholesterol, LDL-C = Low-density lipoprotein cholesterol, HOMA-IR = Homeostasis model assessment of insulin resistance, FBS = Fasting blood glucose.

## 10. Conclusions

This narrative review highlights the role of oxidative stress in the pathogenesis of diabetes in the groups of menopause transition and male-onset hypogonadism. Maintaining high antioxidant capacity offers much to women and men undergoing hormonal transitions, as it alleviates metabolic consequences and improves health span trajectories. As a “take-home message”, leading an antioxidant lifestyle and seeking the appropriate antioxidant supplementation is a good practical recommendation in clinical practice. However, due to the antioxidant paradox, future studies are necessary to further elucidate the role of antioxidant supplementation as a clinical approach to prevent the metabolic consequences of reproductive hormones’ withdrawal.

Maintaining a healthy and antioxidant lifestyle may be essential for preventing the metabolic and cardiometabolic consequences of menopause and andropause. Physical activity and plant-based diet types are well-established measures to assist a smooth transition in menopause and andropause [78].

## Figures and Tables

**Table 1:** Summary of selected meta-analyses on antioxidant supplementation.

Antioxidant	Study (Year)	Study Type	No. of Studies	Key Findings
Resveratrol	Mousavi et al. (2019) [52]	SR/MA	28	Reduce BW, BMI and WC, but not FM
Resveratrol	Setayesh et al. (2026) [53]	SR/MA	23	Not significantly improve BW, BMI, FM, adiponectin and leptin levels. Modest reduction in WC.
Resveratrol	García-Martínez et al. (2022) [54]	SR/MA	15	Dose- and age- dependent decrease of glucose concentrations, HbA_1c_, and insulin levels
Resveratrol	Zhu et al. (2024) [55]	MA	6	Reduced oxidative stress and inflammation in T2DM patients to a certain degree
Vitamin E	Karimi et al. (2025) [56]	SR/MA	10	No overall effect; subgroup benefits in NAFLD
Vitamin C	Mason et al. (2021) [57]	SR/MA	28	Improve glycemic control and BP with moderate to very low evidence certainty
Vitamin C + E	Aragón-Vela et al. (2025) [58]	SR/MA	52	Reduction in SBP with vitamin C and vitamins C + E. HDL improved with combination
Alpha-lipoic acid	Namazi et al. (2018) [59]	SR/MA	12	Small reductions in weight and BMI
Alpha-lipoic acid	Vajdi et al. (2020) [60]	SR/MA	18/21/8 *	Reduced BW and BMI; WC dependent on duration
Alpha-lipoic acid	Luo et al. (2025) [61]	SR/MA	11	No significant associations with TG, TC, HDL-C, LDL-C, HOMA-IR and FBS levels
Alpha-lipoic acid	Salinas et al. (2026) [62]	SR/MA	15	Improved neuropathic symptoms; no glycemic effect

* Different outcomes included different numbers of studies.

## Data Availability

No new data were created or analyzed in this study. Data sharing is not applicable to this article.

## Abbreviations

The following abbreviations are used in this manuscript:

ABP	Androgen-binding protein
Akt	Protein kinase B
BMI	Body mass index
BW	Body weight
CRP	C-reactive protein
FM	Fat mass
FSH	Follicle-stimulating hormone
GH	Growth hormone
GLP-1	Glucagon-like peptide-1
GLUT-4	Glucose transporter-4
GnRH	Gonadotropin-releasing hormone
GPx	Glutathione peroxidase
hCG	Human chorionic gonadotropin
HDL	High-density lipoprotein
HOMA-IR	Homeostatic Model Assessment of Insulin Resistance
HPT	Hypothalamus–pituitary–testicular
IGF-1	Insulin-like growth factor-1
IL-1β	Interleukin-1 beta
IL-6	Interleukin-6
IRS	Insulin receptor substrate
JNK	c-Jun N-terminal kinase
LDL	Low-density lipoprotein
LH	Luteinizing hormone
LOH	Late-onset hypogonadism
MA	Meta-analysis
MAPK	Mitogen-Activated Protein Kinase
MDA	Malondialdehyde
NAC	N-acetylcysteine
NAFLD	Nonalcoholic fatty liver disease
NO	Nitric oxide
NOS	Nitric oxide synthase
PI3K	Phosphatidylinositol 3-kinase
RNS	Reactive nitrogen species
ROS	Reactive oxygen species
SBP	Systolic blood pressure
SOD	Superoxide dismutase
SR	Systematic review
StAR	Steroidogenic acute regulatory protein
T2DM	Type 2 diabetes mellitus
TC	Total cholesterol
TG	Triglyceride
TNF-α	Tumor necrosis factor-alpha
TRT	Testosterone replacement therapy
WC	Waist circumference
